# Synthesis of Temperature Sensing Nitrogen-Doped Carbon Dots and Their Application in Fluorescent Ink

**DOI:** 10.3390/molecules28186607

**Published:** 2023-09-14

**Authors:** Pingping Liu, Lu Ga, Yong Wang, Jun Ai

**Affiliations:** 1 Inner Mongolia Key Laboratory of Environmental Chemistry, College of Chemistry and Environmental Science, Inner Mongolia Normal University, 81 Zhaowudalu, Hohhot 010022, China; 15049125664@163.com; 2 College of Pharmacy, Inner Mongolia Medical University, Jinchuankaifaqu, Hohhot 010110, China; 13404832082@163.com; 3 College of Geographical Science, Inner Mongolia Normal University, 81 Zhaowudalu, Hohhot 010022, China

**Keywords:** carbon dots, nitrogen, fluorescent ink, temperature

## Abstract

With the discovery of research, many properties of carbon dots are getting better and better. People have taken advantage of this and utilized them interspersed in various fields. In the present study, water-soluble nitrogen-doped carbon dots (N-CDs) with excellent optical and fluorescence thermal properties were prepared by the hydrothermal method using 4-dimethylaminopyridine and *N*,*N*′-methylenebisacrylamide as precursors. Co^2+^ has a selective bursting effect on the fluorescence of N-CDs. The fluorescence of N-CDs is selectively burst by Co^2+^, and the high sensitivity is good in the range of 0–12 μM with a detection limit of 74 nM. In addition, the good temperature response (reversible and recoverable fluorescence in the temperature range of 20~90 °C) and excellent optical properties of the N-CDs also make them new potentials in the field of fluorescent inks and temperature sensing.

## 1. Introduction

Cobalt exerts its good physicochemical properties in many fields, such as batteries, electrocatalysis, and photocatalysis [1,2,3,4]. And in biology, cobalt is an important micronutrient, and as one of the metal ions necessary for the formation of vitamin B complexes in the body, it is involved in the regulation of various body functions. However, if Co^2+^ accumulates in excess in the body, it can lead to a variety of diseases [5,6,7].

The current detection methods for Co^2+^ are surface-enhanced Raman scattering [8], electrochemical methods [9], and colorimetric methods [10]. These techniques require detection conditions (equipment, cumbersome procedures, or pre-treatment) that are difficult to meet in many areas. This severely limits the real-time determination of Co^2+^ and its content in practical applications.

With unique optical properties, low toxicity, and biocompatibility, carbon dots have many applications in optics, energy, and biomedicine [11,12,13]. As a small (typically less than 10 nm) carbon-based nanomaterial, it is expected to replace other conventional fluorescent nanomaterials in the development of a high-performance probe. Recently, Huang and his team [14] used a polymer encapsulated with carbon dots to monitor Cu^2+^ in the endoplasmic reticulum (ER), while the carbon dots prepared by Yang et al. targeted the ER and significantly eliminated superoxide and hydroxyl radicals [15]. In addition, the carbon dots can selectively detect a variety of other metal ions (e.g., Zn^2+^, Mg^2+^, Ag^+^, and Fe^3+^) [16,17,18,19]. The high specificity for the target substance during the experiment, simplicity, and speed of analysis make them ideal for Co^2+^ detection [20,21,22].

Temperature is a fundamental thermodynamic variable, and its accurate determination is very important. The reason for this is that the temperature of diseased cells in the organism is higher than that of normal cells. As a result, there is now an increasing demand for accuracy in the measurement of temperature, which is very important for the detection and treatment of diseases. Carbon dots are slowly coming into the limelight because of their good sense of temperature [23,24,25,26].

**Scheme 1:**
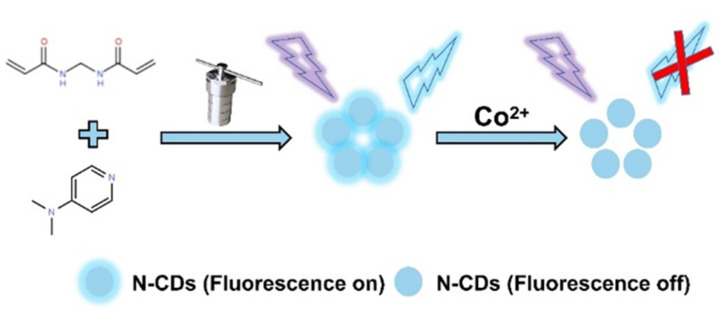
Method for synthesizing N-CDs and sensors for detecting Co^2+^.

Various studies have shown that doping with N atoms can effectively modulate and change the intrinsic properties of carbon dots [27]. 4-dimethylaminopyridine is a novel and efficient catalyst, and *N*,*N*′-methylenebisacrylamide is an indispensable cross-linker and condenser in the polymer field. In this paper, 4-Dimethylaminopyridine and *N*,*N*′-methylenebisacrylamide were used for the first time as precursors for the preparation of N-CDs. We directly used two N-containing substances as precursors to increase the nitrogen content. The prepared N-CDs have better photostability and FL performance without further modification (Scheme 1). Experimental data on Co^2+^ recovery in real samples indicate that the method proposed in this paper is reliable and feasible for Co^2+^ determination in water samples. The bright blue fluorescence of N-CDs remains stable after prolonged UV light exposure or high salt concentrations. The fluorescence intensity of N-CDs decreased with increasing temperature in the range of 20~90 °C, with a good linear relationship. The fluorescence intensity was maintained at 91.76% after multiple heating and cooling steps. This reversibility and recoverability make it possible to enter the field of temperature sensing. Our study not only enriches research on heteroatom-doped carbon dots but also explores new reaction precursors for subsequent research on nitrogen-doped carbon dots.

## 2. Results and Discussion

### 2.1. Preparation of N-CDs

Using 4-dimethylaminopyridine and *N*,*N*′-methylenebisacrylamide as raw materials, N-CDs were prepared by a hydrothermal method. The reaction raw materials were optimized based on fluorescence intensity (supporting information Figure S1a,b). The fluorescence intensity is highest when the mass ratio of 4-dimethylaminopyridine to *N*,*N*′-methylenebisacrylamide is 0.4 g:0.3 g (reaction at 220 °C for 16 h).

### 2.2. Characterization

**Figure 1:**
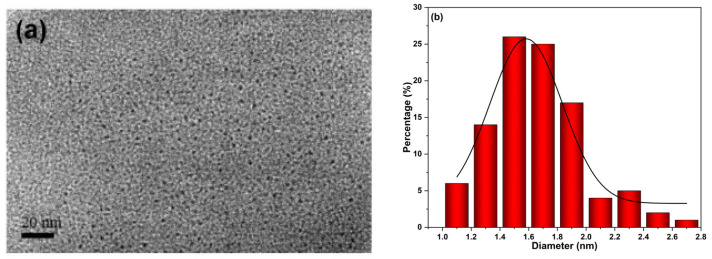
(**a**) TEM images and (**b**) size distribution of N-CDs.

N-CDs were spherical and well dispersed, with a particle size distribution ranging from 1.07 to 2.76 nm and a mean particle size of 1.66 nm, as shown by transmission electron microscopy (TEM) images (Figure 1a). The broad peak around 20.8° in the XRD spectrum (Figure S2a) is showing the amorphous carbon structure of N-CDs [28]. 

The Fourier transform infrared spectroscopy (FT-IR) plot (Figure S2b) shows that the peak tensile vibration of the O–H/N–H bond is located at 3432 cm^−1^ [18]. And the peak at 2932 cm^−1^ originates from the =C–H stretching vibration [29]. The presence of C=O, C=C, and C–N is confirmed by three peaks at 1642 cm^−1^, 1450 cm^−1^, and 1374 cm^−1^ [28,30]. In addition, it may also contain C–O–C (1108 cm^−1^). The presence of these groups allows us to prepare N-CDs with good hydrophilicity and stability in water.

**Figure 2:**
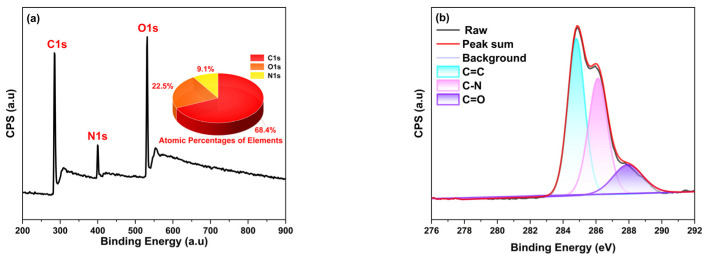

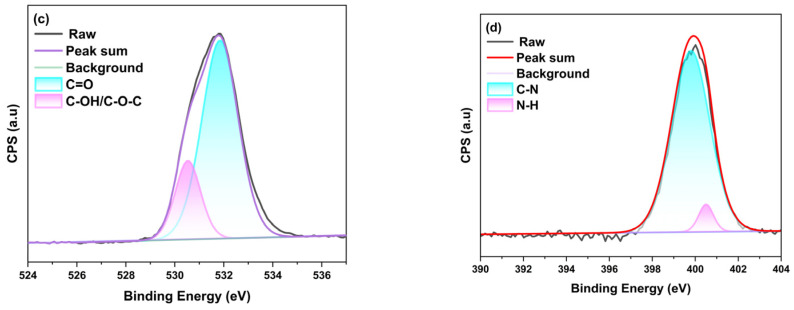
(**a**) XPS spectra of N-CDs. (**b**) C1s. (**c**) O1s. (**d**) N1s spectrum of the N-CDs.

From the XPS spectra, it can be seen that the N-CDs mainly contain three elements: carbon (284.7 eV, 68.36%), nitrogen (399.7 eV, 9.14%), and nitrogen (531.7 eV, 22.50%) (Figure 2a). In the high-resolution XPS spectra, the three peaks of C1s (284.8 eV, 286.1 eV, and 287.9 eV) represented the presence of C=C, C–N, and C=O, respectively (Figure 2b). The N1s spectra indicated the presence of N–H (399.8 eV) and C–N (400.5 eV) (Figure 2c). The two peaks in the O1s spectrum confirm the presence of C=O and C–OH/C–O–C (Figure 2d) [31]. 

### 2.3. Optical Properties

The results of UV-Vis absorption spectroscopy tests performed on it (Figure S3a) showed that an absorption peak near 215 nm could be attributed to a π-π* leap in the C=C bond [32,33,34]. In contrast, the absorption near 345 belongs to the n-π* leap of C=O/C=N [35,36]. In addition, Figure S3b shows the strong emission (λem = 487 nm) under optimal excitation (λex = 414 nm). The prepared N-CDs emit bright cyan fluorescence under 365 nm UV light. Figure S3c shows that the emission wavelength of N-CDs changes its position and intensity (the position is red-shifted and the intensity increases and then decreases) with the increase of the excitation wavelength (Figure S3d), indicating that the position of the emission peaks and the fluorescence intensity are both related to the change of the position of the excitation wavelength. This phenomenon is caused by surface defects, i.e., uneven particle size of N-CDs or uneven distribution of different functional groups on the surface of N-CDs [37].

### 2.4. The Stability of the N-CDs

The strong photobleaching resistance of N-CDs was demonstrated by the fact that their N-CDs fluorescence intensity remained essentially unchanged under UV light (365 nm) for 80 min (Figure S4a,b). The fluorescence intensity also remained essentially unchanged between 0.1 and 0.5 M NaCl concentrations, indicating that N-CDs are stable at high salt concentrations (Figure S4c). From Figure S4d, we can see that 4 min is the optimal reaction time. This is because after the addition of N-CDs to the Co^2+^ solution, although the burst effect can be achieved at 0.5 min (a significant reduction from the highest intensity of 1 to 0.38), it basically stabilizes after 4 min.

### 2.5. Detection of Co^2+^ and Method Selectivity

**Figure 3:**
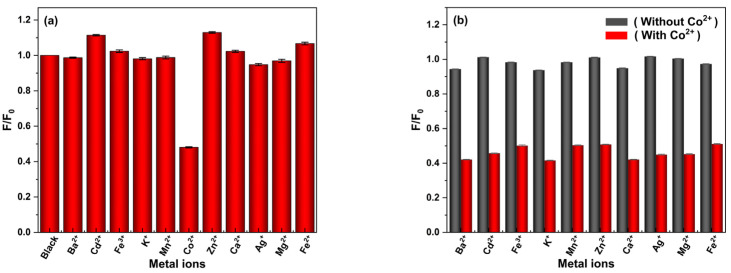
Fluorescence response of N-CDs to (**a**) different metal ions. (**b**) Fluorescence response when Co^2+^ and other metal ions coexist.

The high selectivity for Co^2+^ is essential for N-CDs. In order to test its practicality in real samples, experiments were conducted to determine the selectivity and interference immunity of this sensor. The response of N-CDs to different metal ions (Ba^2+^, Cd^2+^, Fe^3+^, K^+^, Mn^2+^, Zn^2+^, Ca^2+^, Ag^+^, Mg^2+^, and Fe^2+^) at a concentration of 0.01 M was investigated. In Figure 3a, only Co^2+^ induces a marked reduction in the fluorescence intensity of N-CDs at 487 nm. This indicates that N-CDs have a high specificity for Co^2+^. This phenomenon may be related to the fact that the surface functional groups (containing nitrogen or oxygen) of the prepared N-CDs are negatively charged and have a high affinity for Co^2+^ ions [36]. In addition, the addition of Co^2+^ to a system in which N-CDs coexist with other metal ions has a very significant effect on the coexisting system. The fluorescence intensity of N-CDs only decreased significantly when Co^2+^ coexisted with other metal ions, which suggests that the detection of Co^2+^ by N-CDs is not affected by the presence of other metal ions (Figure 3b). In addition, we found that some metal ions can cause insignificant fluorescence enhancement in N-CDs. This may be due to the chelation of functional groups on the surface of N-CDs with metal ions to form complexes, which improve photoinduced electron transfer and lead to enhanced emission [38].

### 2.6. Detection Limit of Co^2+^

**Figure 4:**
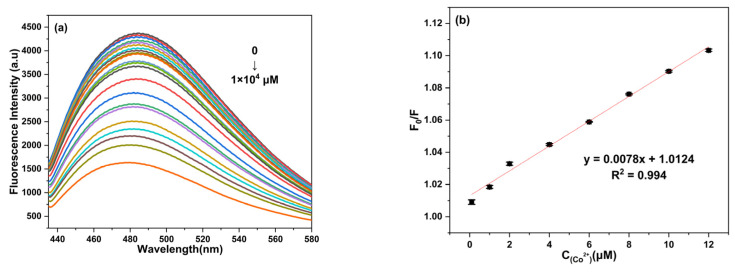
(**a**) Emission spectra of N-CDs after the addition of different amounts of Co^2+^. (**b**) Relationship between (F_0_/F) and Co^2+^ concentration (F_0_ and F represent the fluorescence intensity of N-CDs without and with the addition of Co^2+^, respectively).

As seen in Figure 4a, the fluorescence intensity of N-CDs near 487 nm decreases regularly (in the range of 0.1–1 × 10^4^ μM) with a gradual increase in the concentration of added Co^2+^. Figure 4b shows a good linear relationship (R^2^ = 0.994) between the concentration of Co^2+^ solution (0–12 μM) and the fluorescence intensity. The calculated result of LOD is 74 nM (3σ/s) (the calculation process of LOD is provided in ESM).

As an important parameter for assessing the analytical performance of Co^2+^ monitoring methods, a lower limit of detection (LOD) for Co^2+^ represents better analytical performance and higher sensitivity for the substance. Compared with many currently reported carbon-spot-based probes for the determination of Co^2+^ (Table S1), the LOD values of the N-CDs prepared in this thesis were superior to those reported for the detection of Co^2+^, indicating that the carbon-spots prepared here successfully achieved high sensitivity for the determination of Co^2+^.

### 2.7. Possible Detection Mechanisms

**Figure 5:**
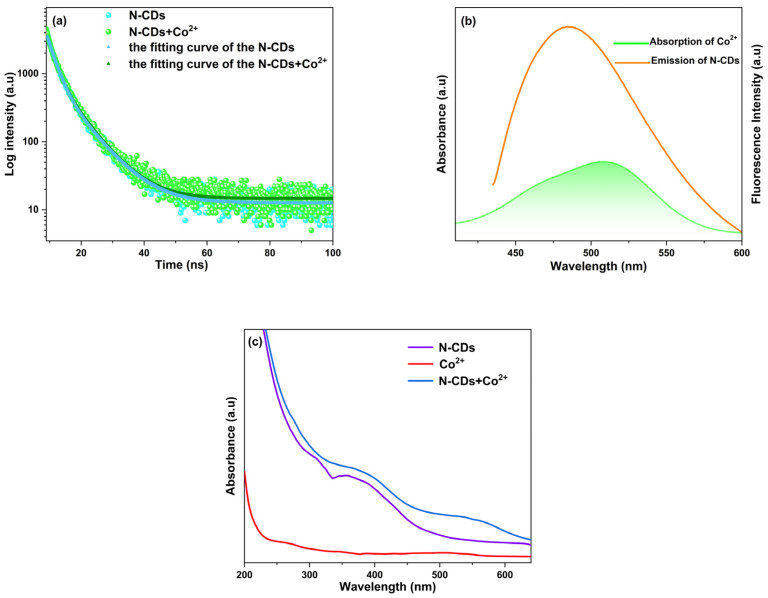
(**a**) Comparison of fluorescence lifetimes. (**b**) Overlay of N-CDs emission spectra and Co^2+^ UV-Vis absorption spectra. (**c**) UV-Vis absorption spectra of Co^2+^ and N-CDs (with and without added Co^2+^).

In order to investigate the mechanism by which Co^2+^ quenches the fluorescence of N-CDs, the possible fluorescence quenching mechanisms were analyzed in this paper using the standard Stern–Volmer equation, fluorescence lifetime curves, UV-vis absorption spectra, and fluorescence spectra. In Figure S5, Co^2+^ ions (0–12 μM) showed a good linear relationship with F/F_0_. A *Ksv* value of 0.007 μM^−1^ can be derived from the standard Stern–Volmer equation. The addition or absence of Co^2+^ had little effect on the average fluorescence lifetime of N-CDs (the lifetime decayed from 5.03 ns to 5.01 ns) (Figure 5a). This suggests that the bursting mechanism is unlikely to be dynamic quenching (DQE). In addition, the presence of the internal filtering effect (IFE) was demonstrated by the overlap of the emission spectra of N-CDs with the absorption peak of Co^2+^ in the 428 to 600 nm range (Figure 5b). The formation of a non-fluorescent complex between Co^2+^ and N-CDs resulted in a new absorption peak near 550 nm (Figure 5c). And this is proof of the existence of the static burst effect (SQE). The final analysis showed that Co^2+^ could quench the fluorescence of the N-CDs prepared in this paper based on the SQE and IFE mechanisms. This is in agreement with the literature reports [37,39].

### 2.8. Detection of Co^2+^ in Real Samples

Therefore, the results of Co^2+^ content determined from actual water samples (supermarket pure water) using the standard additive recovery method were 96.32–100.17% recovery of Co^2+^ with a relative standard deviation (RSD) of 2.65–4.42% (<5%). This implies that N-CDs are one of the ideal methods for the determination of low levels of Co^2+^ in a quantitative assay environment (Table S2).

### 2.9. Research on Temperature Sensing Performance of N-CDs

**Figure 6:**
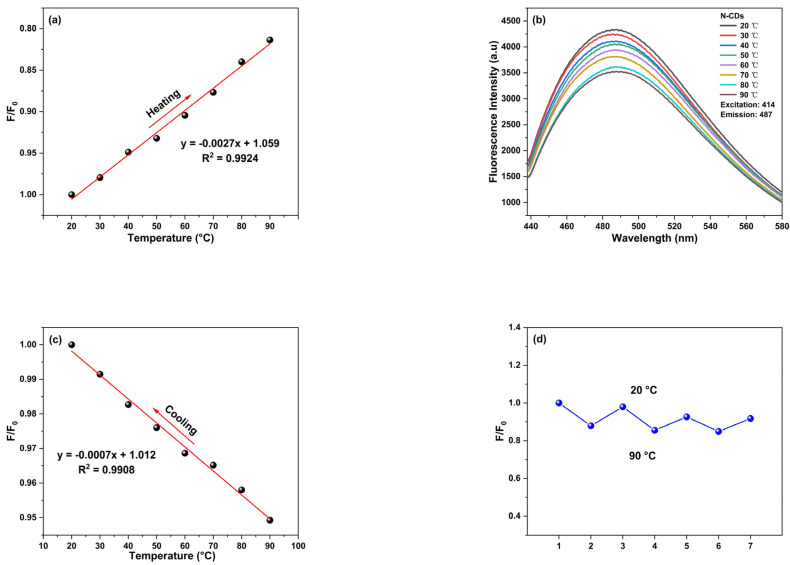
(**a**) Changes in fluorescence intensity of N-CDs during heating (20~90 °C). (**b**) Emission spectra of N-CDs at different temperatures. (**c**) Changes in fluorescence intensity of N-CDs during cooling (20~90 °C). (**d**) Reversible temperature fluorescence of N-CDs.

N-CDs are temperature-sensitive by measuring their fluorescence intensity over a temperature range of 20~90 °C. The temperature from 20 °C to 90 °C was measured in 10 °C temperature gradients. The decrease in fluorescence intensity with increasing temperature can be seen in Figure 6b. The increase in temperature causes the non-radiative channels to be activated, causing more excited electrons to return to the ground state via non-radiative jumps, resulting in a decrease in fluorescence intensity [40,41,42]. It was also found that the value of (F/F_0_) decreases with increasing temperature (R^2^ = 0.9924) and increases with decreasing temperature (R^2^ = 0.9908) (Figure 6a,c). Moreover, in this temperature range (20~90 °C), no migration of the emission peak positions of N-CDs with temperature was observed to occur (Figure 6b). The reusability was tested by recording the fluorescence intensity of N-CDs in three cycles of heating (90 °C) and cooling (20 °C) (Figure 6d). Finally, its maximum fluorescence intensity was still maintained at 91.76%, a result that indicates that its fluorescence center was not destroyed, and the good linear properties and reusability of the fluorescence of N-CDs ensure its potential application in the field of temperature sensing.

### 2.10. Application of N-CDs in the Field of Fluorescent Ink

**Figure 7:**
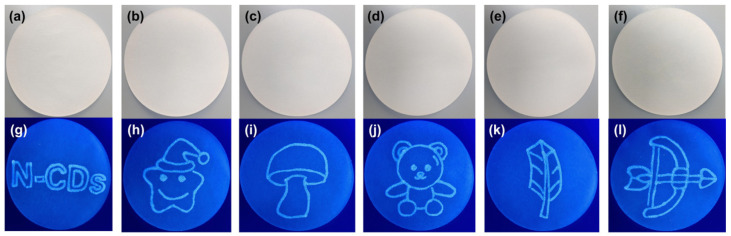
Photographs of N-CDs drawn with fluorescent ink taken in daylight (**a**–**f**) and (**g**–**l**) under UV irradiation.

In addition to fluorescence sensing by Co^2+^ and temperature sensing, the N-CDs proposed in this paper have been shown to emit bright cyan fluorescence and are therefore applicable to the field of fluorescent inks. We found that fluorescent inks have excellent optical properties and can be used for information storage. Some letters and drawings were written on filter paper, as shown in Figure 7. Figure 7a–f were taken in daylight. Figure 7g–l were taken under ultraviolet light (365 nm). The UV irradiation showed a bright cyan fluorescence. The fluorescence properties of these filter papers were not affected after several months of storage at room temperature, indicating the excellent fluorescence stability of our N-CDs.

## 3. Experimental Instruments

### 3.1. Materials and Apparatus

The materials and instruments used in this work are listed in the Electronic Support Materials (ESM).

### 3.2. Preparation of N-CDs

The detailed procedure for preparation is given in the supporting information.

### 3.3. Detection of Co^2+^ and Interference Experiments

Before recording the fluorescence emission spectra (excitation wavelength 414 nm), a series of solutions of known Co^2+^ content were added to the aqueous solution of N-CDs and allowed to react for 4 min.

Similarly, in selective experiments, other common metal ions (K^+^, Fe^2+^, Fe^3+^, Hg^2+^, Ba^2+^, Ni^2+^, Cu^2+^, Zn^2+^, Mn^2+^, Ca^2+^) were added to the testing system instead of Co^2+^, and the changes in F_0_/F were recorded while other experimental conditions remained unchanged (λex/λem = 414 nm/487 nm).

## 4. Conclusions

In conclusion, in this paper, N-CDs were synthesized using 4-dimethylaminopyridine and *N*,*N*′-methylenebisacrylamide as precursors. The detection limits and linear ranges of N-CDs as fluorescent probes for Co^2+^ by fluorescence quenching were 74 nM and 0–12 μM, respectively. Compared with the existing studies for the detection of Co^2+^, this study not only provides a new raw material for the preparation of N-CDs but also shows that the method is suitable for the detection of Co^2+^ in real samples. In addition, the N-CDs have potential applications in fluorescent inks in addition to their reversible and recoverable fluorescence properties at temperature, which expands their application range.

## Data Availability

Not applicable.

## Supplementary Materials

The following supporting information can be downloaded at: https://www.mdpi.com/article/10.3390/molecules28186607/s1 [43,44,45,46,47]. Figure S1. (a) Fluorescence emission spectra of N-CDs prepared from reaction materials with different mass ratios. (b) Changes in fluorescence intensity with different mass ratios of reaction materials; Figure S2. (a) X-ray diffraction and (b) FT-IR of N-CDs; Figure S3. N-CDs (a) UV visible absorption spectra. (b) fluorescence spectra (Illustration: Image of N-CDs under natural light (left) and ultraviolet light (365 nm) (right) irradiation). (c) fluorescence spectra at different excitation wavelengths. (d) Normalized spectra of fluorescence spectra at different excitation wavelengths; Figure S4. (a) Fluorescence spectra of N-CDs under 80 min ultraviolet lamp irradiation and (b) Line chart. (c) The effect of NaCl solution concentration on N-CDs. (d) The intensity of N-CDs varies with reaction time after the addition of Co^2+^ (F_0_ and F represent the fluorescence intensity of N-CDs at 487 nm in the presence and absence of Co^2+^, respectively); Figure. S5. Stern Volmer relationship between (F/F_0_) and Co^2+^ concentration; Table S1: Comparison of the performance of synthesized N-CDs with other N-CDs in Co^2+^ detection; Table S2: Determination of Co^2+^ in purified water samples.

## Sample Availability

Not available.
