# Supplementary material for: Synthesis of Temperature Sensing Nitrogen-Doped Carbon Dots and Their Application in Fluorescent Ink
Source: Molecules. 2023 Sep 14;28(18):6607. doi: 10.3390/molecules28186607 (PMC10536200; doi:10.3390/molecules28186607)
Supplement: Supplementary file 1 [file molecules-28-06607-s001.zip › molecules-2553305-supplementary.pdf]

Supporting Information

Synthesis of Temperature Sensing Nitrogen-Doped Carbon Dots and Their Application in Fluorescent Ink

The water used in the experiments was ultrapure water (18.2 MΩ·cm) prepared by Milli.Q ultrapure water system. The main experimental reagents were 4-dimethylaminopyridine ($C_7H_{10}N_2$); N,N'-Methylenebisacrylamide ($C_7H_{10}N_2O_2$); NaCl; KCl; $FeCl_2·4H_2O$; $FeCl_3·6H_2O$; $HgCl_2$; $CoCl_2·6H_2O$; $BaCl_2·2H_2O$; $NiCl_2·6H_2O$; $CuCl_2·2H_2O$; $ZnCl_2$; $MnCl_2·4H_2O$; $CaCl_2$. All the above reagents are analytically pure.

Apparatus

JEM-2100F transmission electron microscope (JEOL, Japan) was utilized to record the transmission electron microscopy (TEM) of N-CDs. Fluorescence spectra were obtained by a F97pro type fluorescence spectrometer (Shanghai, China). Ultraviolet-visible absorption spectra (UV-vis) were obtained by a U-2900 UV-visible absorption spectrometer (Hitachi High-Technologies Corporation). The instruments used in the preparation were centrifuge model D1008 (Dragon Laboratory Instruments Limited), constant temperature magnetic heating stirrer HJ-4A (Jiangsu Jintan Honghua Instrument Factory), intelligent ultrasonic cleaner DL-108D (Shanghai Zhisun Equipment Co. (Shanghai Zhisun Equipment Co., Ltd), DF-101S constant temperature water bath (Henan Yuhua Instrument Co., Ltd), DHG-9015A blast dryer (Shanghai Yiheng Technology Instrument Co. Ltd.). Fourier transform infrared spectroscopy (FT-IR) recorded on a Nicolet iS20 (Thermo Scientific).

Preparation of N-CDs

The N-CDs were synthesized by a facile hydrothermal method. first, 0.4 g of 4-dimethylaminopyridine and 0.3 g of N,N'-Methylenebisacrylamide were dissolved in 16.0 mL of ultrapure water by sonication, and the mixture was poured into a 50 ml stainless steel Teflon-lined autoclave, placed in an oven and heated continuously at 220 °C for 16 h. After heating and cooling to room temperature, the solution was centrifuged, After cooling to room temperature, the solution was centrifuged (12000 rpm) for 15 min and the initial product was filtered through a membrane (0.22 μm). The obtained N-CDs solution was dialyzed in ultrapure water using a dialysis bag (molecular retention=1000)($C_{N\text{-}CDs}$=2 mg/mL). Finally, the solution was stored at 4 °C for backup.

Detection of Co^{2+} in real samples

The actual water sample was a pure water from a supermarket, and the water sample was filtered with a disposable microporous membrane (0.22 μm) to remove the particulate matter and insoluble matter from the water sample. The spiked sample was made by adding a certain amount of Co^{2+} standard solution to the actual water sample, and the concentration of Co^{2+} in the spiked sample was obtained by using the standard addition method.

Calculation of detection limit (LOD)

The calculation formula for the detection limit (LOD) is as follows:

LOD=3 σ/s

among σ is the standard deviation of the blank, and *s* is the slope of the linear fitting curve.

Quenching constant (Ksv) calculation

To investigate possible quenching mechanisms, the standard Stern-Volmer equation was used to describe the relationship between quenching signals and Co^{2+}concentration [1], as follows:

$$F_0/F = 1 + Ksv[Q]$$

Among them, F_0 and F represent the fluorescence intensity without and with Co^{2+}, respectively, [Q] is the concentration of Co^{2+}, and *Ksv* is the quenching constant.

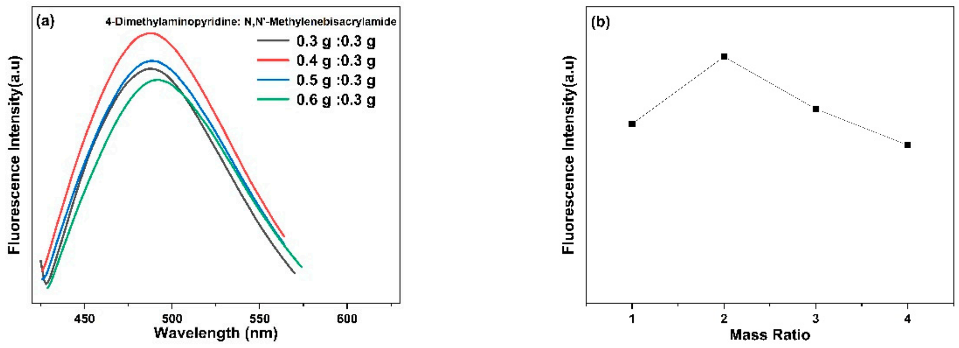

Figure S1. (a) Fluorescence emission spectra of N-CDs prepared from reaction materials with different mass ratios. (b) Changes in fluorescence intensity with different mass ratios of reaction materials.

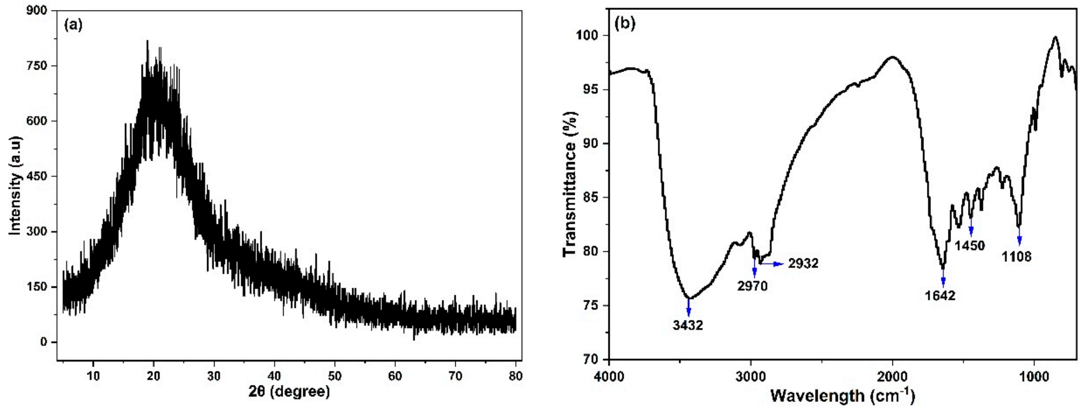

Figure S2. (a) X-ray diffraction and (b) FT-IR of N-CDs.

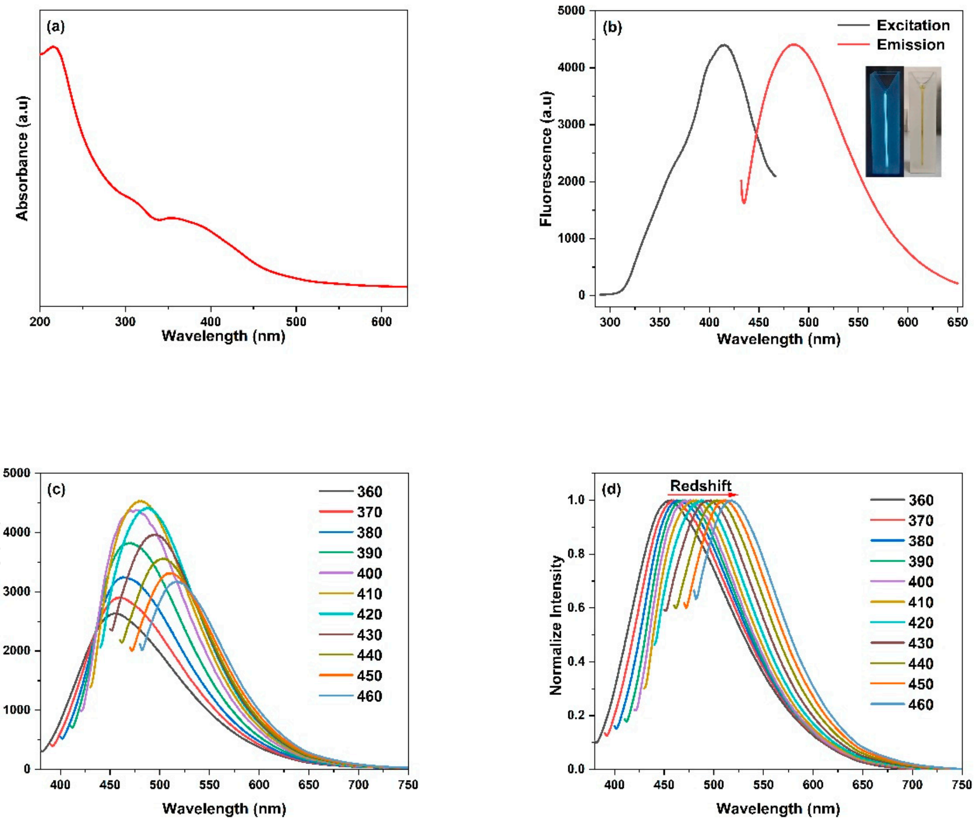

Figure S3. N-CDs (a) UV visible absorption spectra. (b) fluorescence spectra (Illustration: Image of N-CDs under natural light (left) and ultraviolet light (365 nm) (right) irradiation). (c) fluorescence spectra at different excitation wavelengths. (d) Normalized spectra of fluorescence spectra at different excitation wavelengths.

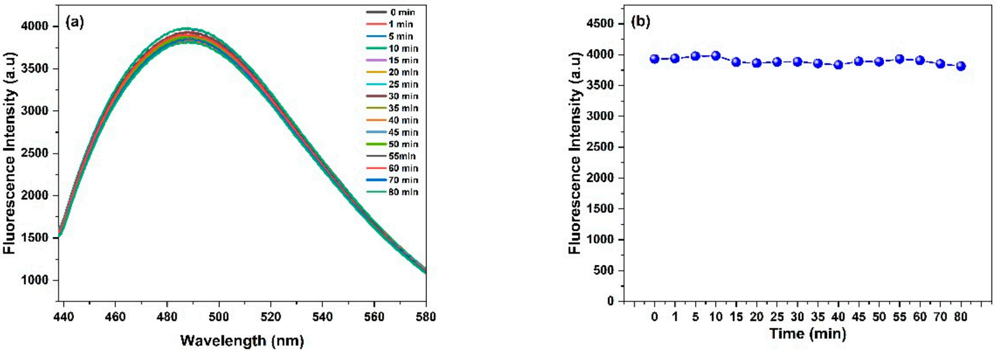

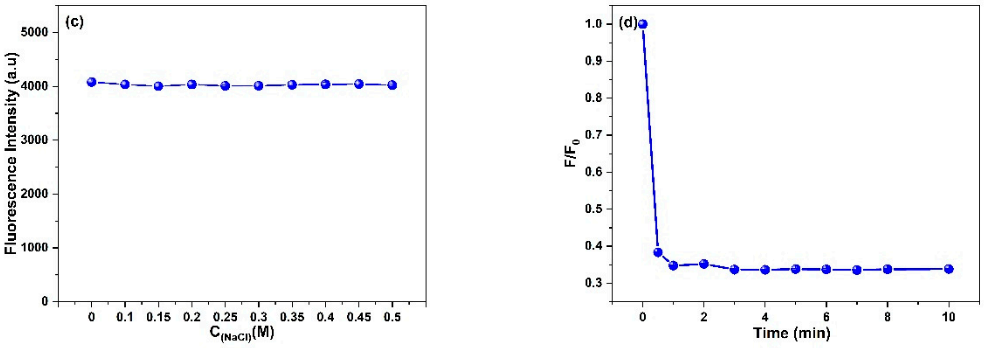

Figure S4. (a) Fluorescence spectra of N-CDs under 80 min ultraviolet lamp irradiation and (b) Line chart. (c) The effect of NaCl solution concentration on N-CDs .(d) The intensity of N-CDs varies with reaction time after the addition of Co^{2+}(F_0 and F represent the fluorescence intensity of N-CDs at 487 nm in the presence and absence of Co^{2+}, respectively).

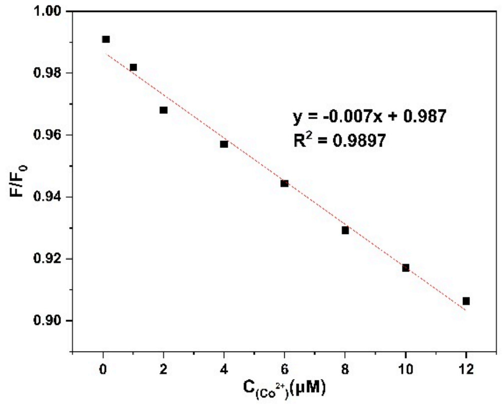

Figure S5. Stern Volmer relationship between (F/F_0) and Co^{2+}concentration.

Table S1. Comparison of the performance of synthesized N-CDs with other N-CDs in Co^{2+}detection.

**Precursor**	**Sensing Platform**	**Linear Range (μM)**	**LOD**	**Ref.**
Acrylic acid , ethylenediamine	Nitrogen-doped carbon dots (NCDs)	1.0-60 μM	0.25μM	[2]
Citric acid, Cysteamine hydrochloride	Nitrogen and sulfur co-doped graphene quantum dots (N,S-GQDs)	0-40 μM	1.25 μM	[3]
Carbopol 934 , Diethylenetriamine	Carbon dots (CDs)	0 - 40 μM	0.45 μM	[4]

Artemisia annua , 1,2-ethylenediamine	N-doped carbon nanodots (N-CNDs)	2.5-25 μM	230.5 nM	[5]
4-Dimethylaminopyridine, N, N-methylenebisacrylamide	Nitrogen-doped carbon dots (N-CDs)	0 -12 μM	74 nM	this work

Table S2. Determination of Co^{2+}in purified water samples.

Sample	Spiked (μmol/L)	Measured (μmol/L)	Recovery (%)	Relative Standard Deviation (%) (n=3)
1	1	1.04	96.32	3.41
2	4	4.00	99.98	4.42
3	7	6.99	100.17	2.65

1. Liu, S.; Liu, R.; Xing, X.; Yang, C.; Xu, Y.; Wu, D., Highly photoluminescent nitrogen-rich carbon dots from melamine and citric acid for the selective detection of iron(iii) ion. RSC Advances 2016, 6, (38), 31884-31888.
2. Jing, N.; Tian, M.; Wang, Y.; Zhang, Y., Nitrogen-doped carbon dots synthesized from acrylic acid and ethylenediamine for simple and selective determination of cobalt ions in aqueous media. Journal of Luminescence 2019, 206, 169-175.
3. Boonta, W.; Talodthaisong, C.; Sattayaporn, S.; Chaicham, C.; Chaicham, A.; Sahasithiwat, S.; Kangkaew, L.; Kulchat, S., The synthesis of nitrogen and sulfur co-doped graphene quantum dots for fluorescence detection of cobalt(ii) ions in water. Materials Chemistry Frontiers 2020, 4, (2), 507-516.
4. Kong, D.; Yan, F.; Han, Z.; Xu, J.; Guo, X.; Chen, L., Cobalt(ii) ions detection using carbon dots as an sensitive and selective fluorescent probe. RSC Advances 2016, 6, (72), 67481-67487.
5. Du, F.; Cheng, Z.; Kremer, M.; Liu, Y.; Wang, X.; Shuang, S.; Dong, C., A label-free multifunctional nanosensor based on N-doped carbon nanodots for vitamin B_{12} and Co^{2+} detection, and bioimaging in living cells and zebrafish. Journal of Materials Chemistry B 2020, 8, (23), 5089-5095.
